# Needle-Free Devices and CpG-Adjuvanted DNA Improve Anti-HIV Antibody Responses of Both DNA and Modified Vaccinia Ankara-Vectored Candidate Vaccines

**DOI:** 10.3390/vaccines11020376

**Published:** 2023-02-07

**Authors:** Rosamund Chapman, Michiel van Diepen, Nicola Douglass, Tandile Hermanus, Penny L. Moore, Anna-Lise Williamson

**Affiliations:** 1 Institute of Infectious Disease and Molecular Medicine, Faculty of Health Science, University of Cape Town, Cape Town 7925, South Africa; 2 Division of Medical Virology, Department of Pathology, University of Cape Town, Cape Town 7925, South Africa; 3 Centre for HIV and STIs, National Institute for Communicable Diseases of the National Health Laboratory Service, Johannesburg 2131, South Africa; 4 Antibody Immunity Research Unit, Faculty of Health Sciences, University of the Witwatersrand, Johannesburg 2050, South Africa; 5 Centre for the AIDS Programme of Research in South Africa (CAPRISA), University of KwaZulu-Natal, Congella, Durban 4013, South Africa

**Keywords:** HIV vaccine, needle-free, PharmaJet Stratis^®^, DNA vaccine, MVA, CpG

## Abstract

The combination of mosaic Gag and CAP256 envelope in an HIV vaccine regimen comprising DNA prime and modified vaccinia Ankara (MVA) boost followed by protein boost has previously been shown to generate robust autologous Tier 2 neutralizing antibodies (nAbs) in rabbits. Further refinements of this strategy have been investigated to improve antibody responses. The delivery of both DNA and recombinant MVA vaccines with a needle-free device was compared to delivery by injection, and the effect of formulating the DNA vaccine with adjuvant CpG ODN 1826 was determined. The Pharmajet Stratis^®^ needle-free injection device (PharmaJet, Golden, CO, USA) improved binding antibody responses to the DNA vaccine as well as both binding and neutralizing antibody responses to the MVA vaccines. Formulation of the DNA vaccines with CpG adjuvant further improved the antibody responses. A shortened vaccination regimen of a single DNA inoculation followed by a single MVA inoculation did not elicit Tier 1B nor Tier 2 neutralization responses as produced by the two DNA, followed by two MVA vaccination regimen. This study showed the immunogenicity of HIV DNA and MVA vaccines administered in a DDMM regimen could be improved using the PharmaJet Stratis needle-free injection device and formulation of the DNA vaccines with CpG adjuvant.

## 1. Introduction

HIV is still a major problem in the world and particularly in South Africa, where it is estimated that 7.9 million people are living with HIV—which translates to 14% of the population [1]. One of the means of controlling the ongoing HIV pandemic is by prophylactic vaccination; unfortunately, only one trial has shown modest efficacy [2] with a number of recent vaccine trials being unsuccessful [3,4]. Trials of infused monoclonal antibodies demonstrated the broadly neutralizing monoclonal antibody, VRC01, was able to prevent infection by viruses that were sensitive to neutralization by this antibody [5]. Thus, while infusion of VRC01 antibodies did not prevent overall HIV acquisition, it confirmed an HIV vaccine that could induce broad and potent neutralizing antibodies (nAbs) was likely to be protective [6].

There is proof of concept in macaques infused with monoclonal antibodies that if the challenge virus can be neutralized by the antibody the animals are protected [7]. However, the biggest challenge in HIV vaccinology is the genetic variation of the virus making it difficult to select protective antigens that will induce broadly neutralizing antibodies. HIV group M has ten distinct subtypes, A, B, C, D, F, G, H, J, K, and L, as well as various recombinants [8]. The genetic variation of HIV makes the induction of broadly neutralizing antibodies a significant challenge [9,10]. Subtype C has been shown to be the dominant subtype in India, Ethiopia, and Southern Africa. As our vaccines are targeted at South Africa, our strategy is to base vaccine design on HIV subtype C isolates. This would then match the vaccines to the circulating virus subtypes. For most viral vaccines, the induction of neutralizing antibodies that can block infection of the prevalent circulating virus is desirable [11,12,13,14]. However, it also depends on the route of infection and the type of transmission; the age and gender of the person being infected can play a role as well. Different routes of HIV transmission may require different vaccine strategies.

It is not well understood what the correlates of protection are for an HIV vaccine. The one HIV vaccine trial that had some success was RV144. This was a clinical trial in Thailand, which tested a vaccine regimen consisting of a combination of a canary poxvirus-vectored vaccine and protein, ALVAC-HIV+AIDSVAX B/E, AIDSVAX B/E, and ALVAC-HIV, respectively, or placebo. They demonstrated an ALVAC-HIV prime and AIDSVAX B/E boost afforded 60% efficacy against human immunodeficiency virus (HIV) acquisition at 1 year, declining to 31.2% after 3.5 years [15]. In the RV144 trial, the correlate of protection was described as high V1V2 Ab reactivity in plasma and low Env-specific IgA in plasma [16]. However, clinical trials based on the same concept as RV144 have not been successful [3]. In addition, while a lot of emphasis is placed on testing HIV vaccines in non-human primates, the correlates of protection that have been predicted from non-human primate studies as well as various clinical trials are not consistent [17]. This continues to make the field of rational design of HIV vaccines very challenging.

The ideal vaccine response would be antibodies that would prevent HIV infection. This requires antibodies that would neutralize the circulating HIV. However, if this is not possible, the next target would be non-neutralizing, functional antibody responses, such as antibodies that mediate antibody-dependent cellular cytotoxic antibodies (ADCC), a response which has been found to correlate with reduced risk of infection in the RV144 trial [18]. Additionally, T cells that would attack early infected cells before latency was established would perform a similar function to ADCC [19]. T cell responses to HIV Gag have been correlated with viral control; hence, the inclusion of Gag in HIV vaccine candidates where it could impact on early control via CD8+ T-cells at the site of infection, control spread from the entry portal, and control viraemia if infection is established [20]. The early evidence that HIV-specific CD8+ T cell responses in the genital mucosa of HIV-1-resistant sex workers might play an important part in protective immunity against heterosexual HIV-1 transmission gave hope that if this response could be induced by a vaccine, there may be protection from HIV infection [21,22].

Our first-generation vaccines had a natural Gag selected to be closest to the consensus sequence of HIV subtype C viruses sampled in South Africa [23]. This gene, as part of a polyprotein with reverse transcriptases (RTs), Tat and Nef, combined with Env, was tested in clinical trials with a DNA- and MVA-based HIV vaccine boosted with a gp140 protein [24,25]. In these trials, the vaccines did not induce Tier 2 neutralizing antibodies. The response to the polyprotein Gag, RT, Tat, and Nef (grrttn) was skewed in favor of Pol. Based on these results, a next generation vaccine was designed with an improved Gag and Env. In mice, we demonstrated a mosaic HIV subtype C Gag [26] induced significantly higher CD8+ responses than the natural Gag that had been in our original vaccine, and thus, our vaccines now contain this mosaic Gag [27].

An envelope sequence was chosen from a virus isolated from a patient in the South African CAPRISA 002 acute infection cohort, patient CAP256, who developed broadly neutralizing antibodies (bnAbs) following a secondary infection of HIV-1 approximately 15 weeks after the primary infection [28]. The CAP256 superinfecting viral envelope was selected as the donor-developed high titre, broadly neutralizing responses that were particularly potent against HIV subtypes, C and A viruses [29]. Vaccines expressing a combination of mosaic Gag and CAP256 Env in a regimen comprising two DNA primes and two modified vaccinia Ankara (MVA) boosts followed by two gp140 protein boosts gave robust HIV Tier 2 nAbs against the autologous virus from CAP256 in rabbits [30].

To further enhance this response, we investigated the impact of administering the DNA and MVA vaccines using two different strategies. In the first modification, we used the PharmaJet Stratis needle-free device, which has been used successfully to administer MVA vaccines against HIV [31] and smallpox [32,33] and inactivated influenza [34] and polio vaccines [35] and DNA vaccines against Zika [36], Hantavirus [37], and Venezuelan encephalitis virus [38]. Secondly, we assessed the addition of an adjuvant, which can enhance specific immune responses [39,40]. In our study, DNA vaccines were formulated with the CpG adjuvant oligonuceotides (ODN) 1826. CpG ODN stimulates Toll-like receptor 9 (TLR9), which is expressed on human plasmacytoid dendritic cells and B cells. Activation of TLR9 induces an innate Th1 immune response [41]. CpG adjuvant ODN 1826 has been reported to enhance mouse immune responses elicited by an HIV DNA vaccine targeting Gag and gp120 [42]. It has also been tested with a peptide-based mucosal HIV vaccine [43].

## 2. Materials and Methods

### 2.1. Vaccines

**Figure 1:**
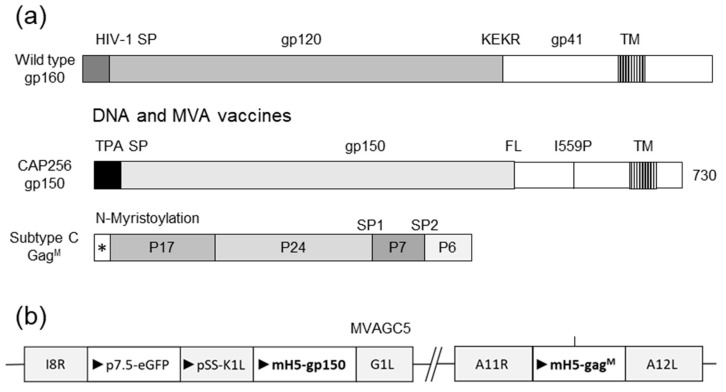
Design of vaccines utilized in this study. (**a**) Wild type envelope (gp160), modified gp150, and subtype C mosaic Gag used in the DNA and MVA vaccines. HIV-1 SP = native signal peptide; KEKR = furin cleavage site; TM = transmembrane domain; TPA SP = tissue plasminogen signal peptide; FL = flexible linker; I559P = isoleucine to proline mutation at amino acid 559; * = N-myristoylation site; SP1 = spacer region 1; SP2 = spacer region 2. (**b**) Schematic representation of genes inserted into MVA vaccine. I8R-G1L and A11R-A12L indicate the loci of the insertions in MVA. Triangles indicate the direction of open reading frames.

The HIV Env sequence used in the vaccines was derived from CAP256.SU gp160 (clone CAP256.206sp.032.C9) [44] and was modified as follows: the native leader sequence was replaced with the human tissue plasminogen activator (TPA) leader sequence, the furin cleavage site was replaced with a flexible linker sequence (FL) [45], and an I548P mutation equivalent to the I559P in the SOSIP trimers was introduced to promote trimerization of gp41 [46]. Finally, the sequence was truncated to gp150 (amino acid 730) to increase expression and stability. The Env sequence was human codon optimized and synthesized by GenScript (Nanjing, China) (Figure 1) [30]. DNA and MVA vaccines expressing CAP256 gp150 and subtype C mosaic Gag, as shown in Figure 1a, were used [30]. The design of the MVA vaccine is shown in Figure 1b.

### 2.2. Rabbit Immunisations

Female New Zealand white rabbits (age ± 10 weeks, weight ≥ 2.2kg) were housed in the animal facility of the Faculty of Health Sciences at the University of Stellenbosch. Groups of five rabbits were used. All the animal procedures were approved by the UCT Animal Research Ethics Committee (reference UCT AEC 015–051 and 019–015) and performed by trained animal technologists. DNA and MVA vaccines were administered intramuscularly into the hind leg at 100 µg (100 µL of each) and 10^8^ pfu (500 µL), respectively. The DNA vaccine consisted of two plasmids, formulated together in equal quantities; the first expressed the HIV-1 CAP256 gp150 envelope protein and the second the subtype C mosaic Gag (100 µg of each). DNA and MVA vaccines were administered by needle injection or with the PharmaJet Stratis (PharmaJet, Golden, CO, USA) device as indicated. DNA vaccines were administered with and without CpG adjuvant ODN 1826 (27.5 μg/rabbit) (Miltenyi Biotec, Bergisch Gladbach, Germany).

### 2.3. Anti-Env Enzyme-Linked Immunosorbent Assays (ELISA) and HIV Neutralisation Assays

Env-binding antibody titres in the rabbit sera were determined as previously de-scribed [30]. In short, Nunc MaxiSorp^®^ flat-bottom 96 well plates (Sigma, St. Louis, MO, USA) were coated with 10 ng/well soluble, trimeric CAP256 Env. Rabbit sera were used in the primary incubation in a serial dilution range starting at 1:10. Anti-rabbit IgG HRP (1:5000, Roche) was used for detection with TMB ELISA substrate (Abcam, Cambridge, UK). The reaction was stopped after 10 min with 1N H_2_SO_4_. The ELISA signal was analyzed using a VersaMax ELISA Microplate Reader (Molecular Devices), which subtracted absorbance values at 540 nm from values at 450 nm. ELISAs for the whole time course and each group were performed at the same time on duplicate plates. Duplicate data points were averaged and fitted to a four-parameter logistic regression curve (4 PL curve) in GraphPad Prism 5.0. Antibody end-point titres were calculated from 4 PL curves with the threshold set as 4 PL curve minimum + standard error of minimum for each time point. Data was plotted as the mean ± SEM for the whole group.

The standardized TZM-bl pseudovirus neutralization assay, was used to determine neutralizing antibody titres as follows. Neutralization was measured as a reduction in luciferase gene expression after a single round of infection of JC53bl-13 cells, also known as TZM-bl cells (NIH AIDS Research and Reference Reagent Program), with Env-pseudotyped viruses. Titre was calculated as the reciprocal plasma/serum dilution causing a 50% reduction of relative light units (ID50). Dilutions were started at 1:20. For graphs, data was plotted as 19 when ID50 was <20. MuLV was used as the negative control.

### 2.4. Statistical Analysis

All statistical analysis was performed using GraphPad Prism 5.0 (San Diego, CA, USA). Mann–Whitney testing and two-way ANOVA were performed with Bonferroni post hoc testing.

## 3. Results

### 3.1. PharmaJet Stratis Needle-Free Injection Device Improves Immune Responses

**Figure 2:**
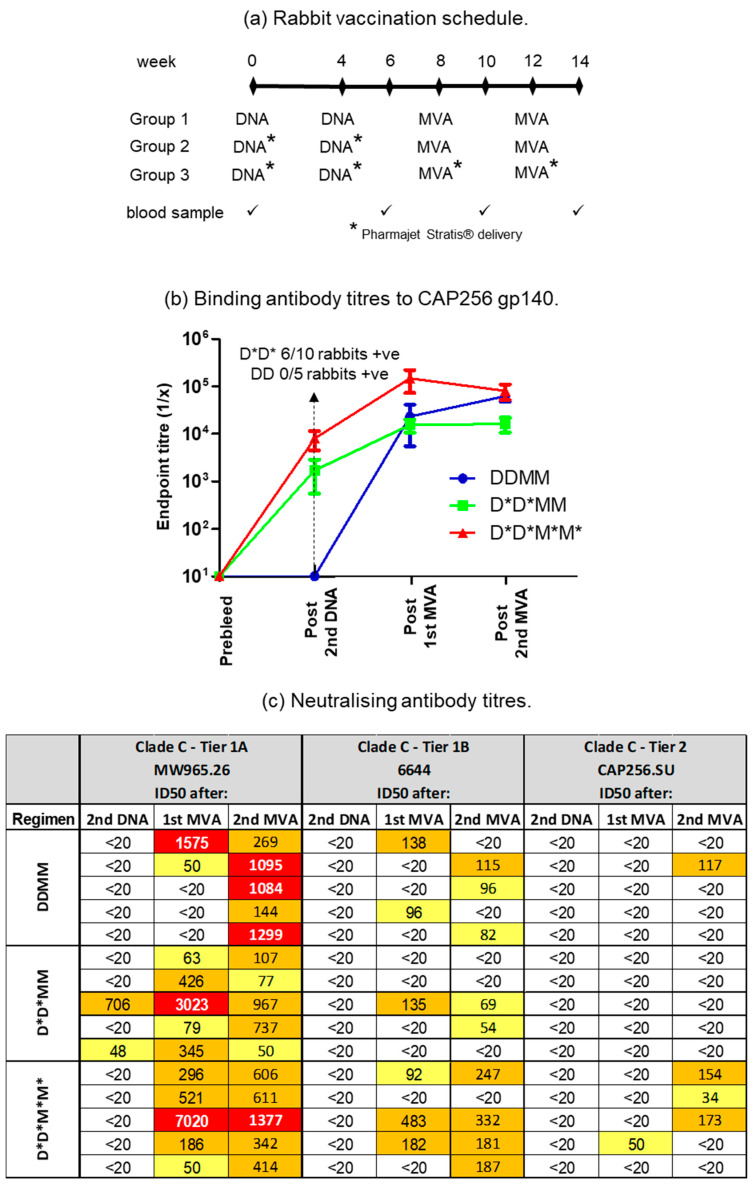
Comparison of the use of the PharmaJet Stratis device for the delivery of DNA and MVA vaccines. (**a**) Rabbit vaccination schedule for the three different groups. * = use of the PharmaJet Stratis device. (**b**) Binding antibody titres to CAP256 gp140 in sera of vaccinated rabbits. When no binding was observed, the end point titre was plotted as 10. (**c**) Neutralizing antibody titres in rabbit sera measured using the TZM-bl assay. The 50% neutralization titres were color-coded as follows: yellow = 20–100; orange = 100–1000; red = 1000–10,000. Titres below 20 were considered negative. D = DNA; M = MVA.

**Figure 3:**
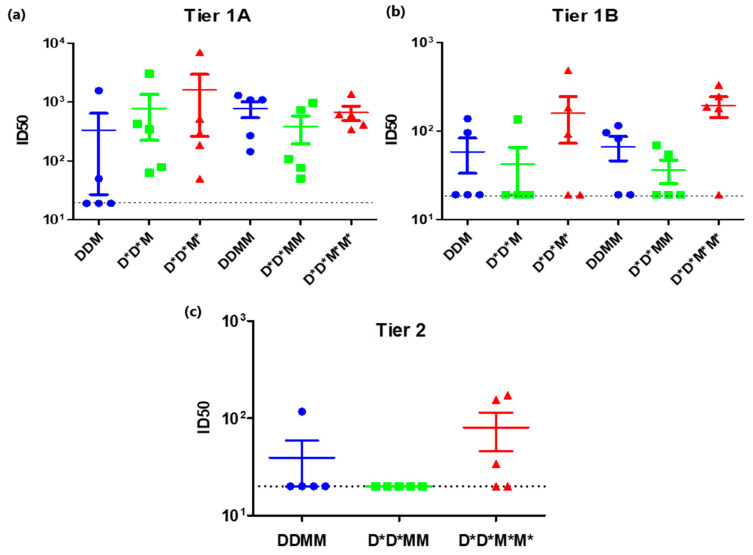
A comparison of Tier 1A, Tier 1B, and Tier 2 neutralizing antibody responses elicited after inoculation using the PharmaJet Stratis device. (**a**) Tier 1A and (**b**) Tier 1B neutralizing antibody titres in rabbit sera after 2 DNA and 1 MVA inoculation (DDM) and after 2 DNA and 2 MVA inoculations (DDMM). (**c**) Tier 2 neutralizing antibody titres in rabbit sera after 2 DNA and 2 MVA inoculation (DDMM) The dotted black line represents the assay detection limit (1/20 dilution). Data were plotted as the mean ± SEM. * = vaccine was inoculated using the PharmaJet Stratis device.

Use of the PharmaJet Stratis needle-free injection device for delivery of DNA- and MVA-based vaccines was compared to inoculation with a needle and syringe (Figure 2). Rabbits were given two doses of the DNA vaccines, followed by two doses of the MVA vaccines. All the vaccines were administered to Group 1 using a needle and syringe. Group 2 animals were given DNA vaccines using the PharmaJet Stratis needle-free injection device and MVA vaccines using needle and syringe. Group 3 animals received both the DNA and MVA vaccines via needle-free injection. The needle-free injection device clearly improved immune responses to the DNA vaccine. Six of the ten rabbits inoculated with the PharmaJet Stratis needle-free injection device (Groups 2 and 3) developed binding antibody responses after the second DNA vaccination, whereas none of the rabbits that received the DNA vaccines via needle injection (Group 1) developed binding antibody responses (Figure 2b). Inoculation with the PharmaJet Stratis needle-free injection device also improved responses to the MVA vaccination as three out of the five rabbits in Group 3 developed neutralizing antibody responses to the Tier 1B pseudovirus 6644 after the first MVA vaccination, as compared to only one rabbit in Group 2 (Figure 2c and Figure 3). Administration of the DNA and MVA vaccines with the PharmaJet Stratis device also showed a trend towards increasing mean titres of neutralizing antibodies. Group 3 animals that received both the DNA and MVA vaccines via needle-free injection developed Tier 2 nAbs with a mean ID_50_ of 80.2, as compared to the rabbits in Group 1 that received both vaccines via needle and syringe, which had a mean ID_50_ of 39. In addition, three out of the five rabbits in Group 3 developed autologous Tier 2 neutralizing antibody responses (nAbs) to pseudovirus CAP256SU (mean ID_50_ 80.2), whereas none of the Group 2 rabbits (mean ID_50_ 20) and only one of the Group 1 rabbits (mean ID_50_ 39) developed Tier 2 nAbs. There was also a definite trend towards increased mean titres of Tier 1A nAbs from Groups 1 to 3 after the first MVA inoculation with the use of the PharmaJet device (mean titres 336.4 DDM, 787.2 D*D*M, and 1614.6 D*D*M* or geometric mean titres 55.8 DDM, 294.4 D*D*M, and 398.6 D*D*M*. * = use of Pharmajet Stratis device). Although there were clear differences in the numbers of responding rabbits and a definite trend towards higher antibody titres with the use of the needle-free injection device, statistical analysis showed no significant differences in the titres (Figure 3).

### 3.2. A Shortened Vaccination Regimen Elicits Inferior Neutralizing Antibody Responses

**Figure 4:**
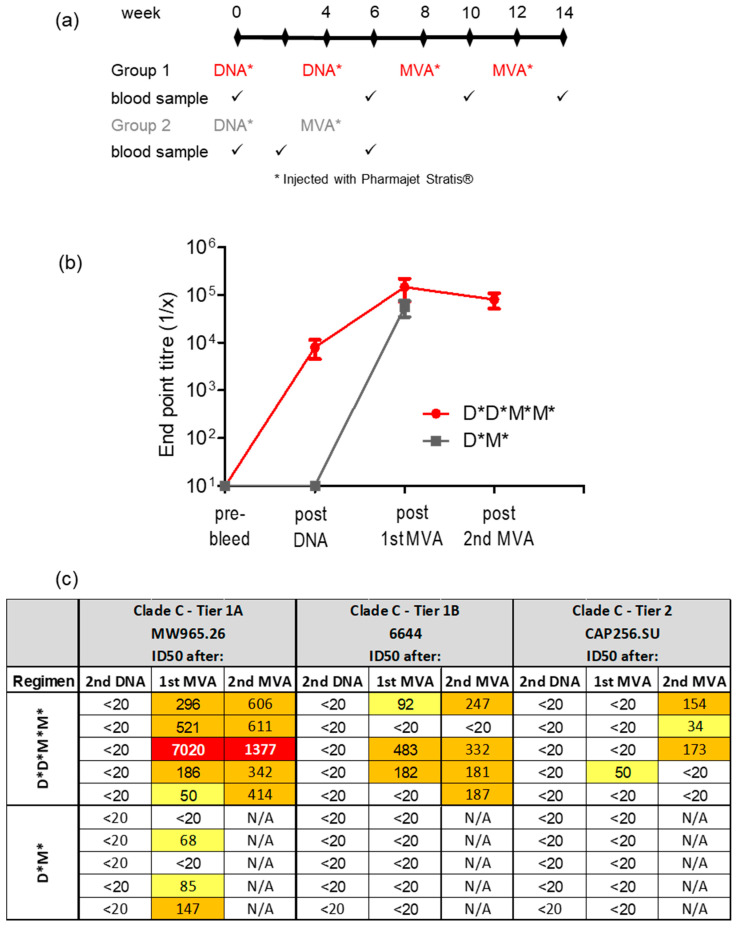
Antibody responses elicited in rabbits inoculated with a shortened vaccination regimen. (**a**) Rabbit immunization protocol. All vaccines were administered with the PharmaJet Stratis device. (**b**) Time course showing binding ELISA for rabbit sera. When no binding was observed, the end point titre was plotted as 10. (**c**) Neutralizing antibody titres in rabbit sera measured using the TZM-bl assay. The 50% neutralization titres were color-coded as follows: yellow = 20–100; orange = 100–1000; red = 1000–10,000. Titres below 20 were considered negative. * = vaccine was inoculated using the PharmaJet Stratis device. D = DNA; M = MVA.

As delivery of the DNA and MVA vaccines using the PharmaJet Stratis had improved the immune response elicited, we next sought to reduce the number of inoculations from 2× DNA followed by 2× MVA to 1× DNA, followed by 1× MVA (Figure 4a). Binding antibody responses elicited using the shortened regimen were similar to those of the longer regimen following MVA inoculation (Figure 4b). However, no Tier 1B nAbs were seen after a single DNA and MVA inoculation. In comparison, for the longer vaccination regimen three out of five rabbits developed Tier 1B nAbs, one out of five developed Tier 2 nAbs after the first MVA inoculation, and three out of five developed Tier 2 nAbs after the second MVA inoculation (Figure 4c).

### 3.3. Administering DNA Vaccines with CpG Adjuvant ODN 1826 Improves Antibody Responses

**Figure 5:**
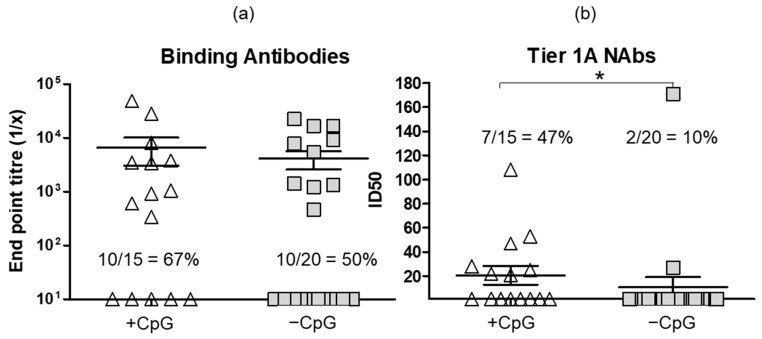
The effect of administering DNA vaccines with CpG adjuvant ODN 1826 on binding and Tier 1A neutralizing antibody responses. The DNA vaccines were formulated with CpG (15 rabbits) or without CpG (20 rabbits) and delivered using the PharmaJet Stratis device at zero and four weeks. Immune responses were assessed two weeks after the second DNA inoculation. (**a**) Binding anti-body titres in sera of rabbits. When no binding was observed, the end point titre was plotted as 10. (**b**) Tier 1A neutralizing antibody responses. The numbers and percentages on the graphs indicate the number of rabbits that developed antibody responses. * *p* = 0.025

In an attempt to further enhance the immune response, the DNA vaccines were formulated with the CpG adjuvant ODN 1826 and delivered with the PharmaJet Stratis device. The antibody responses induced in rabbits given DNA vaccines with and without CpG were then compared two weeks after the second DNA inoculation. Administering the DNA vaccines with CpG led to an increase in the numbers of rabbits that developed binding antibodies (67% versus 50%, not significantly different) and Tier 1A neutralizing antibodies (47% versus 10%, *p* = 0.0255 Mann–Whitney test) (Figure 5). No Tier 2 nAbs and only very low levels of Tier 1B nAbs were detected in two rabbits (ID_50_ 33 and 38) after two DNA inoculations with CpG. However, none of the rabbits that received DNA without CpG developed either Tier 1B or Tier 2 nAbs after two DNA inoculations.

## 4. Discussion

The vaccines in this study have been tested previously, and it was shown that rabbits that received two DNA primes followed by two modified vaccinia virus Ankara (MVA) and two protein inoculations developed better immune responses than those that received two MVA and three protein inoculations. In addition, DNA and MVA vaccines that expressed mosaic Gag VLPs presenting a stabilized Env antigen elicited better responses than Env alone, supporting the inclusion of Gag VLPs in an HIV-1 vaccine [30]. A study was also done in mice where immunogenicity of the HIV-1 subtype C mosaic Gag (Gag^M^) was compared to a naturally occurring subtype C Gag from HIV-1 Du422 (Gag^N^) using a DNA homologous vaccination regimen. Two vaccinations with a DNA vaccine expressing Gag^M^ induced cumulative HIV-1 Gag-specific IFN-gamma ELISPOT responses that were 6.5-fold higher than those induced by a DNA vaccine expressing Gag^N^ [27].

In the present study, different strategies were investigated to improve the antibody responses to the candidate HIV vaccines. The first was delivery of the DNA- and the MVA-based HIV vaccines with a needle-free device, and the other was to investigate the impact of adjuvant CpG ODN 1826 on the DNA vaccine. In addition, the possibility of shortening the vaccination regimen was investigated.

The needle-free jet injection developed by PharmaJet, Inc. can deliver vaccines using a narrow, precise fluid stream, which is an improvement over the standard needle and syringe [38]. The PharmaJet Stratis needle-free injection device improved immune responses to the DNA vaccine, with six of the ten rabbits having binding antibody responses after the second DNA vaccination, whereas none of the rabbits that received the DNA vaccines via needle injection developed binding antibody responses. There was also an improvement in both binding and neutralization responses after the MVA vaccines were administered using the PharmaJet Stratis device as compared to needle injection. Three out of five rabbits receiving both DNA and MVA via the PharmaJet Stratis device had detectable Tier 2 neutralizing antibodies (mean ID_50_ 80.2) compared with only one animal (mean ID_50_ 39.2) in the group receiving these vaccines by injection.

These results concur with other studies that have shown the immunogenicity of DNA vaccines to be enhanced with PharmaJet Stratis delivery as compared to needle and syringe [36,37,38,47,48]. This study also demonstrated significant improvement in the antibody responses with the MVA vaccine delivered using this device.

While herologous prime boost strategies are not unusual in HIV vaccinology, it is desirable not to have a regime that is too complex. Therefore, we decided to test one DNA followed by one MVA immunization. However, significantly better responses were elicited using the regimen of two DNA followed by two MVA vaccinations as compared to only one of each. It is possible these responses could be improved by further boosting with an HIV protein-based vaccine in an appropriate adjuvant.

The inclusion of CpG adjuvant ODN 1826 in the DNA vaccines also improved antibody responses. There was an improved Tier 1A neutralizing antibody response after one DNA inoculation (47% versus 10%, *p* = 0.0255 Mann–Whitney test). Although there was a positive report on the use of this adjuvant in 2005 for a DNA-based HIV vaccine [42], the only other HIV vaccine tested using this adjuvant is one based on a peptide [43]. The use of this adjuvant has been reported to have improved immune responses to other DNA vaccines, for example against schistosomiasis [49], toxoplasmosis [50], atherosclerosis [51], various bacteria [52,53], and tumors [54,55,56].

The challenge of developing an effective HIV vaccine has led researchers to investigate many diverse strategies. This study shows how small modifications in vaccine administration can contribute towards improved immunogenicity. These findings could be applied to different vaccination strategies, possibly even the relatively recent mRNA approach. Different modes of vaccine administration (needle-free vs. needle injection) as well as the use of adjuvants (CpG ODN 1826) could inform future clinical trials.

## 5. Conclusions

The immunogenicity of DNA- and MVA-based vaccines against HIV subtype C viruses can be improved by using the PharmaJet Stratis needle-free injection device inducing Tier 2 neutralizing antibodies. In addition, the CpG adjuvant ODN 1826, when administered together with DNA vaccines, improves responses to Tier 1 HIV isolates.

## 6. Patents

The recombinant MVA described in this paper is covered by a provisional patent “Recombinant MVA with Modified HIV-1 Env”, Patent Application Number PCT/IB2018/057731, filed on 4th October 2018 (GB 1716181.1 filed on 4 October 2017). The inventors are Anna-Lise Williamson, Edward Peter Rybicki, Michiel van Diepen, Nicola Jennifer Douglass, and Rosamund Eira Chapman.

## Data Availability

Not applicable.
